# *Syn* and *anti* conformers of di­ammonium aqua­bis(malonato)oxidovanadate(IV) in an anhydrate crystal

**DOI:** 10.1107/S2056989018005686

**Published:** 2018-04-17

**Authors:** Keiji Ohno, Takumi Yoshida, Akira Nagasawa, Takashi Fujihara

**Affiliations:** a Department of Chemistry, Graduate School of Science and Engineering, Saitama University, Shimo-Okubo 255, Sakura-ku, Saitama 338-8570, Japan; b Saitama Prefectural Matsuyama Senior High School, 6-10, Matsuyama machi 1-chome, Higashi-Matsuyama, 355-0018, Japan; c Comprehensive Analysis Center for Science, Saitama University, Shimo-Okubo 255, Sakura-ku, Saitama 338-8570, Japan

**Keywords:** mol­ecular structure, crystal structure, oxidovanadate(IV), structural isomers

## Abstract

The asymmetric unit of the title anhydrate compound, (NH_4_)_2_[VO(C_3_H_2_O_4_)_2_(H_2_O)], consists of two independent complex anions and four ammonium cations. In the complex anions, the V^IV^ atoms are each coordinated by two malonate ligands, one water mol­ecule and one oxide O atom in a distorted octa­hedral geometry. The equatorial plane is formed by the malonate O atoms, while the axial positions are occupied by water and oxide O atoms. The difference between the two independent complexes is the relative conformation of the malonate ligands. The two ligands in one complex anion are in a *syn* conformation, while in the other they adopt an *anti* conformation. In the crystal, the complex anions inter­act with the counter-cations and adjacent anions through O—H⋯O, N—H⋯O and C—H⋯O hydrogen bonds. Stacks of alternating layers consisting of either *anti* or *syn* isomers, formed with the aid of the hydrogen bonding, are observed. DFT calculations for the *anti* and *syn* isomers show a similar thermodynamic stability to each other. The crystal used for this analysis was an inversion twin with the ratio of the twin components being 0.270 (13):0.730 (13).

## Abstract 2

In the anhydrate crystal of di­ammonium aqua­bis­(malonato)oxidovanadate(IV), (NH_4_)_2_[VO(C_3_H_2_O_4_)_2_(H_2_O)], two conformers (*syn* or *anti* conformation on the equatorial plane) of complex cations are detected. The DFT calculations for the isomers indicate a slight influence of the conformation on their thermodynamic stability. The anionic complexes inter­act with adjacent anions and counter-cations through hydrogen bonds, and the hydrogen bonds lead to a structure with alternate stacking of layers consisting of either *anti* or *syn* isomers.

## Chemical context   

Dianionic aqua­bis­(malonato)oxidovanadate(IV) has been synthesized with various counter-cations to investigate their structures and magnetic and thermal properties (Tomiyasu *et al.*, 1974 ▸; Pajunen & Pajunen, 1980 ▸; Rocha & Baran, 1988 ▸; Sutradhar *et al.*, 2011 ▸; Sehimi *et al.*, 2016 ▸). Previously, the mol­ecular and crystal structures of di­ammonium aqua­bis(malonato)oxidovanadate(IV) monohydrate were reported (Piro & Baran, 1997 ▸). In the present report, we describe the mol­ecular and crystal structures of the title anhydrate compound.
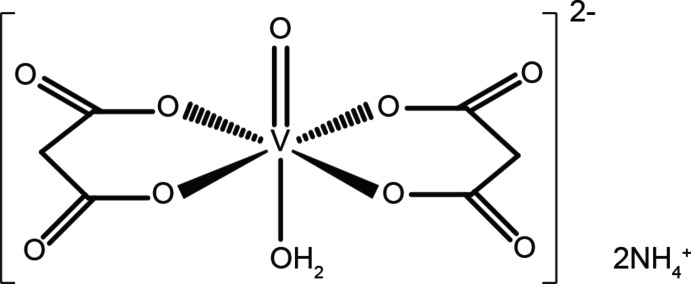


## Structural commentary   

**Figure 1:**
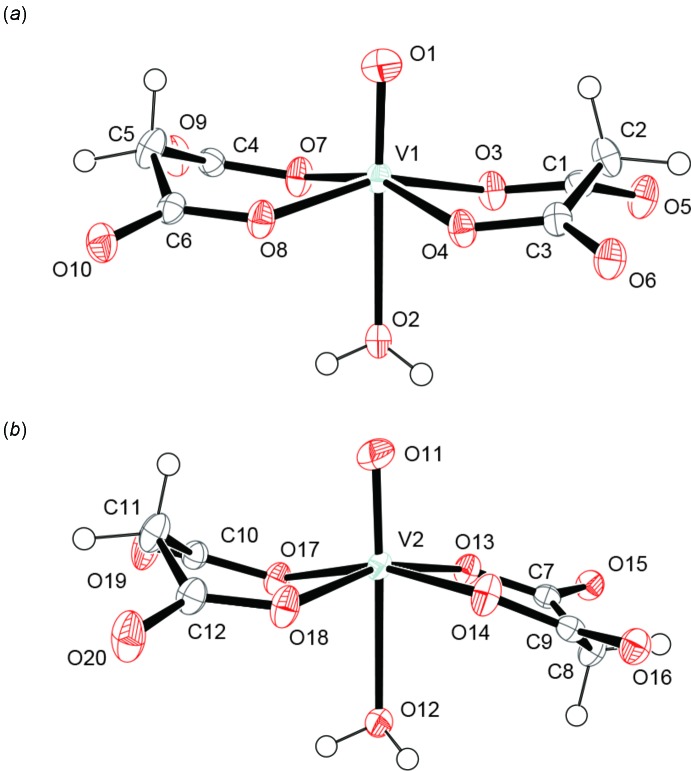
Mol­ecular structures of (*a*) *syn* isomer and (*b*) *anti* isomer. Displacement ellipsoids are drawn at the 50% probability level. The NH_4_^+^ counter-cations have been omitted for clarity.

The asymmetric unit of the title compound contains two crystallographically independent mononuclear complexes and four counter NH_4_^+^ cations. In each complex the water mol­ecule occupies the *trans* position to the oxido O atom, and two malonate ligands coordinate to the V^IV^ center occupying an equatorial plane. Although all six-membered V/O/C–C/O chelate rings in the complexes adopt boat conformations, the whole conformation on the equatorial plane is in either a *syn* conformation or an *anti* conformation (Yuksel *et al.*, 2008 ▸); in the *syn* conformer the two malonate ligands are related to each other by a pseudo twofold rotation axis along the V—O bond, while in the *anti* conformer they are related by an pseudo inversion centre near the V atom (Fig. 1 ▸). The corres­ponding coordination bonds in both conformational isomers show similar distances to each other, and atom V1 in the *syn* isomer and atom V2 in *anti* isomer are located 0.35 and 0.29 Å out of the O3/O4/O8/O7 and O13/O14/O18/O17 planes, respectively. These crystallographic data suggest no influence of the *anti* and *syn* conformations on the coordination geometry around the V^IV^ centre.

Density functional theory (DFT) calculations based on the optimized geometrical parameters were performed at the UB3LYP/6-31G(d) level as implemented in *GAUSSIAN09* (Frisch *et al.*, 2009 ▸). Their structural parameters were extracted from the corresponding X-ray crystallographic data, and the positions of the hydrogen atoms were optimized, while the positions of all other atoms were fixed at their original positions. The results indicate little influence of the conformations on their thermodynamic stability. The calculated sum of electronic and thermal free energies for these isomers show a slight difference (*ca* 11 kJ mol^−1^); the energies of the *anti* and *syn* isomers are −5062702 and −5062713 kJ mol^−1^, respectively.

## Supra­molecular features   

**Figure 2:**
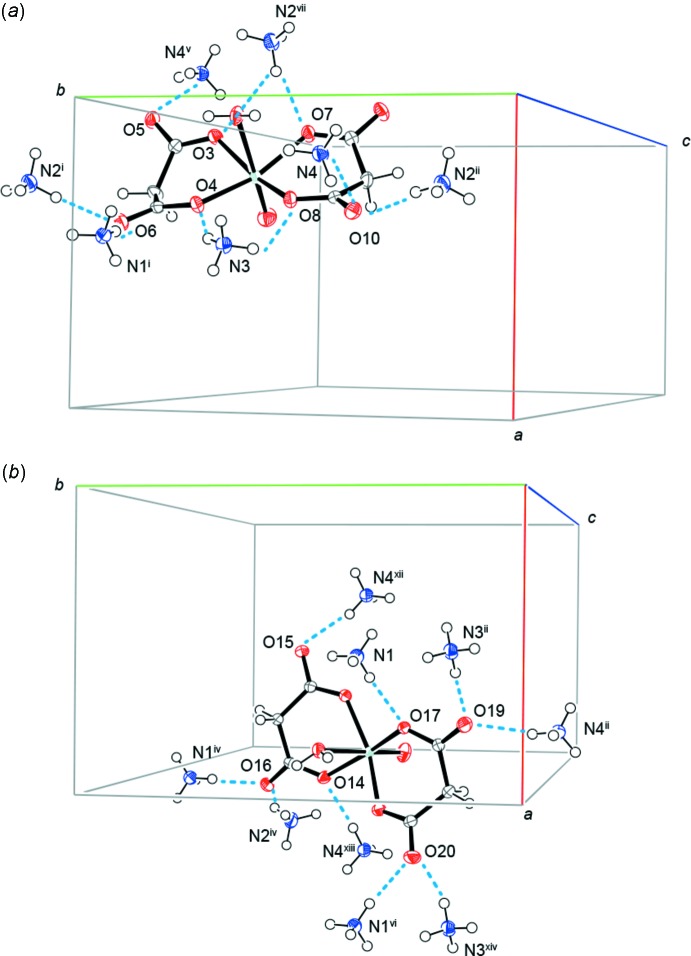
Packing diagrams showing N—H⋯O hydrogen bonds (blue dashed lines) (*a*) between the *syn* isomer and adjacent cations and (*b*) between the *anti* isomer and adjacent cations. Displacement ellipsoids are drawn at the 50% probability level. [Symmetry codes: (i) −*x* + 1, *y* + 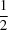, −*z* + 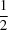; (ii) −*x* + 1, *y* − 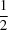, −*z* + 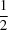; (iv) *x* + 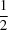, −*y* + 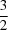, −*z* + 1; (v) −*x*, *y* + 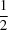, −*z* + 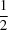; (vi) *x* + 1, *y*, *z*; (vii) *x* − 1, *y*, *z*; (xii) −*x* + 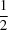, −*y* + 1, −*z* + 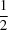; (xiii) −*x* + 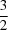, −*y* + 1, −*z* + 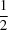; (xiv) −*x* + 2, *y* − 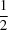, −*z* + 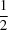.]

**Figure 3:**
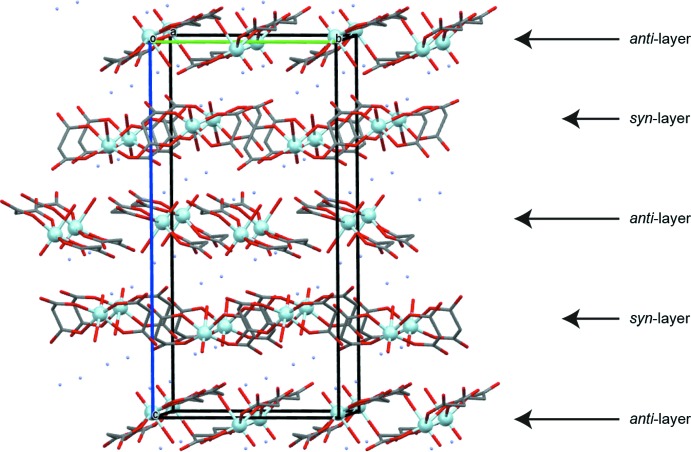
A packing diagram showing the alternating stacking structure. Hydrogen atoms have been omitted for clarity.

The *syn* isomer inter­acts with adjacent seven adjacent ammonium cations *via* N—H⋯O hydrogen bonds and with four other *syn* isomers and one *anti* isomer *via* O—H⋯O and C—H⋯O hydrogen bonds (Table 1 ▸ and Fig. 2 ▸). On the other hand, the *anti* isomer inter­acts with nine adjacent ammonium cations and two *anti* isomers (Fig. 2 ▸). These hydrogen bonds lead to the construction of layers consisting of either *anti* or *syn* isomers expanding parallel to the *ab* plane; the two different layers stack alternately, as depicted in Fig. 3 ▸.

## Synthesis and crystallization   

To an aqueous solution of malonic acid (3.0 g, 29 mmol in 3 ml) was added a concentrated aqueous ammonia solution (0.5 ml). Ammonium metavanadate NH_4_VO_3_ (1.0 g, 8.5 mmol) was added to the solution while it boiled. The solution was then cooled down to room temperature, and EtOH (40 ml) was added to give precipitates. The solution was left standing overnight and then deca­nted to collect the precipitates, which were washed twice with EtOH (20 ml) by stirring followed by deca­ntation. The volume of the resulting blue solution was reduced by heating until crystals appeared. The crude crystals were filtered off at room temperature, dissolved again in water and then ethanol vapor was diffused gradually into the solution. Deep-blue crystals were collected by filtration and dried *in vacuo*. Yield 0.0112 g (0.4% based on NH_4_VO_3_). Analysis found: C 22.04, H 4.28, N 8.32%; calculated for C_6_H_14_N_2_O_10_V: C 22.17, H 4.34, N 8.62%.

## Refinement   

Crystal data, data collection and structure refinement details are summarized in Table 2 ▸. The crystal studied was an inversion twin with a ratio of the twin components of 0.270 (13):0.730 (13). The methyl­ene H atoms were included in calculated positions (C—H = 0.99 Å) and treated as riding atoms with *U*_iso_(H) = 1.2*U*_eq_(C). The H atoms on O and N atoms were located in a difference-Fourier map and refined freely, with restraints of O—H = 0.84 (2) Å and H⋯H = 1.33 (4) Å for the water mol­ecule, and with N—H = 0.84 (2) Å and H⋯H = 1.33 (4) Å for the ammonium ions.

## Supplementary Material

Crystal structure: contains datablock(s) I. DOI: 10.1107/S2056989018005686/is5490sup1.cif

Structure factors: contains datablock(s) I. DOI: 10.1107/S2056989018005686/is5490Isup3.hkl

CCDC reference: 1836312

Additional supporting information:  crystallographic information; 3D view; checkCIF report

## Figures and Tables

**Table 1:** Hydrogen-bond geometry (Å, °)

*D*—H⋯*A*	*D*—H	H⋯*A*	*D*⋯*A*	*D*—H⋯*A*
O2—H2*C*⋯O9^i^	0.84 (2)	2.08 (2)	2.902 (2)	165 (3)
O2—H2*D*⋯O5^ii^	0.82 (2)	1.99 (3)	2.758 (2)	157 (4)
O12—H12*A*⋯O9^iii^	0.85 (2)	1.98 (2)	2.826 (2)	174 (3)
O12—H12*B*⋯O15^iv^	0.84 (2)	2.04 (2)	2.855 (2)	161 (3)
N1—H1*A*⋯O6^v^	0.89 (2)	1.97 (2)	2.833 (2)	164 (2)
N1—H1*B*⋯O20^vi^	0.84 (2)	1.96 (2)	2.787 (2)	168 (3)
N1—H1*C*⋯O16^vii^	0.85 (2)	1.88 (2)	2.711 (2)	164 (2)
N1—H1*D*⋯O17	0.83 (2)	2.02 (2)	2.851 (2)	175 (3)
N2—H2*E*⋯O16^vii^	0.83 (2)	2.01 (2)	2.818 (2)	161 (3)
N2—H2*F*⋯O10^viii^	0.88 (2)	1.96 (2)	2.826 (3)	170 (3)
N2—H2*G*⋯O7^iii^	0.86 (2)	2.18 (2)	3.006 (2)	159 (3)
N2—H2*H*⋯O6^v^	0.87 (2)	2.04 (2)	2.865 (2)	156 (3)
N3—H3*A*⋯O20^ix^	0.82 (2)	2.12 (2)	2.874 (2)	153 (3)
N3—H3*B*⋯O19^viii^	0.84 (2)	1.92 (2)	2.759 (2)	173 (3)
N3—H3*C*⋯O4	0.85 (2)	2.04 (2)	2.828 (2)	153 (3)
N3—H3*D*⋯O8	0.83 (2)	2.33 (3)	2.800 (2)	116 (3)
N4—H4*A*⋯O15^x^	0.85 (2)	1.98 (2)	2.809 (2)	164 (3)
N4—H4*B*⋯O5^ii^	0.85 (2)	2.10 (3)	2.767 (2)	135 (3)
N4—H4*B*⋯O10	0.85 (2)	2.43 (3)	3.105 (2)	136 (3)
N4—H4*C*⋯O14^xi^	0.83 (2)	2.05 (2)	2.872 (2)	169 (3)
N4—H4*D*⋯O19^viii^	0.85 (2)	2.09 (2)	2.885 (2)	156 (3)
C2—H2*B*⋯O10^viii^	0.99	2.59	3.235 (3)	123
C5—H5*A*⋯O6^v^	0.99	2.58	3.275 (3)	127
C5—H5*B*⋯O2^ii^	0.99	2.59	3.432 (3)	143
C8—H8*A*⋯O15^iv^	0.99	2.51	3.207 (2)	127

Symmetry codes: (i) 
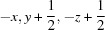
; (ii) 
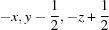
; (iii) 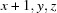; (iv) 
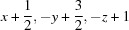
; (v) 
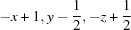
; (vi) 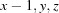; (vii) 
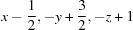
; (viii) 
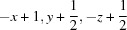
; (ix) 
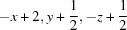
; (x) 
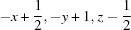
; (xi) 
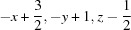
.

**Table 2:** Experimental details

Crystal data	
Chemical formula	(NH_4_)_2_[V(C_3_H_2_O_4_)_2_O(H_2_O)]
*M*_r_	325.13
Crystal system, space group	Orthorhombic, *P*2_1_2_1_2_1_
Temperature (K)	200
*a*, *b*, *c* (Å)	8.3461 (6), 12.1011 (9), 24.2118 (17)
*V* (Å^3^)	2445.3 (3)
*Z*	8
Radiation type	Mo *K*α
μ (mm^−1^)	0.86
Crystal size (mm)	0.15 × 0.13 × 0.12

Data collection	
Diffractometer	Bruker APEXII CCD area-detector
Absorption correction	Multi-scan (*SADABS*; Bruker, 2014 ▸)
No. of measured, independent and observed [*I* > 2σ(*I*)] reflections	13364, 5194, 5080
*R*_int_	0.018
(sin θ/λ)_max_ (Å^−1^)	0.633

Refinement	
*R*[*F* ^2^ > 2σ(*F* ^2^)], *wR*(*F* ^2^), *S*	0.019, 0.053, 1.15
No. of reflections	5194
No. of parameters	424
No. of restraints	32
H-atom treatment	H atoms treated by a mixture of independent and constrained refinement
Δρ_max_, Δρ_min_ (e Å^−3^)	0.25, −0.29
Absolute structure	Refined as an inversion twin.
Absolute structure parameter	0.270 (13)

Computer programs: *APEX2*, *SAINT* *XPREP* and *XCIF* (Bruker, 2014 ▸), *SHELXT* (Sheldrick, 2015*a* ▸), *SHELXL2016* (Sheldrick, 2015*b* ▸) and *ORTEP-3 for Windows* (Farrugia, 2012 ▸).

## supplementary crystallographic information

### Crystal data

**Table:**

(NH_4_)_2_[V(C_3_H_2_O_4_)_2_O(H_2_O)]	*D*_x_ = 1.766 Mg m^−^^3^
*M**_r_* = 325.13	Mo *K*α radiation, λ = 0.71073 Å
Orthorhombic, *P*2_1_2_1_2_1_	Cell parameters from 9961 reflections
*a* = 8.3461 (6) Å	θ = 2.6–28.5°
*b* = 12.1011 (9) Å	µ = 0.86 mm^−^^1^
*c* = 24.2118 (17) Å	*T* = 200 K
*V* = 2445.3 (3) Å^3^	Block, blue
*Z* = 8	0.15 × 0.13 × 0.12 mm
*F*(000) = 1336	

### Data collection

**Table:**

Bruker APEXII CCD area-detector diffractometer	5194 independent reflections
Radiation source: Bruker TXS fine-focus rotating anode	5080 reflections with *I* > 2σ(*I*)
Bruker Helios multilayer confocal mirror monochromator	*R*_int_ = 0.018
Detector resolution: 8.333 pixels mm^-1^	θ_max_ = 26.7°, θ_min_ = 1.7°
φ and ω scans	*h* = −10→7
Absorption correction: multi-scan (SADABS; Bruker, 2014)	*k* = −13→15
	*l* = −30→29
13364 measured reflections	

### Refinement

**Table:**

Refinement on *F*^2^	Hydrogen site location: mixed
Least-squares matrix: full	H atoms treated by a mixture of independent and constrained refinement
*R*[*F*^2^ > 2σ(*F*^2^)] = 0.019	*w* = 1/[σ^2^(*F*_o_^2^) + (0.0322*P*)^2^ + 0.1094*P*] where *P* = (*F*_o_^2^ + 2*F*_c_^2^)/3
*wR*(*F*^2^) = 0.053	(Δ/σ)_max_ = 0.001
*S* = 1.15	Δρ_max_ = 0.25 e Å^−^^3^
5194 reflections	Δρ_min_ = −0.29 e Å^−^^3^
424 parameters	Absolute structure: Refined as an inversion twin.
32 restraints	Absolute structure parameter: 0.270 (13)

### Special details

**Table:**

Geometry. All esds (except the esd in the dihedral angle between two l.s. planes) are estimated using the full covariance matrix. The cell esds are taken into account individually in the estimation of esds in distances, angles and torsion angles; correlations between esds in cell parameters are only used when they are defined by crystal symmetry. An approximate (isotropic) treatment of cell esds is used for estimating esds involving l.s. planes. Least-squares planes (x,y,z in crystal coordinates) and deviations from them (* indicates atom used to define plane) 5.8223 (0.0030) x - 0.2546 (0.0072) y + 17.3398 (0.0086) z = 5.6054 (0.0058) * -0.0205 (0.0008) O3 * 0.0208 (0.0008) O4 * 0.0206 (0.0008) O7 * -0.0209 (0.0008) O8 0.3504 (0.0008) V1 Rms deviation of fitted atoms = 0.0207 0.4684 (0.0044) x - 7.3274 (0.0051) y + 19.2206 (0.0079) z = 6.4071 (0.0078) Angle to previous plane (with approximate esd) = 51.653 ( 0.044 ) * -0.0056 (0.0007) O13 * 0.0059 (0.0008) O14 * 0.0056 (0.0007) O17 * -0.0058 (0.0008) O18 0.2916 (0.0008) V2 Rms deviation of fitted atoms = 0.0057
Refinement. Refined as a 2-component inversion twin.

### Fractional atomic coordinates and isotropic or equivalent isotropic displacement parameters (Å^2^)

**Table:**

	*x*	*y*	*z*	*U*_iso_*/*U*_eq_	
C1	0.1093 (2)	0.97326 (16)	0.31042 (8)	0.0173 (4)	
C2	0.2765 (2)	1.01068 (17)	0.29288 (9)	0.0218 (4)	
H2A	0.278377	1.092433	0.291481	0.026*	
H2B	0.354366	0.987045	0.321386	0.026*	
C3	0.3304 (2)	0.96631 (15)	0.23756 (8)	0.0175 (4)	
C4	0.0831 (2)	0.53339 (15)	0.31370 (8)	0.0178 (4)	
C5	0.2300 (3)	0.48090 (17)	0.28797 (9)	0.0240 (4)	
H5A	0.317370	0.484006	0.315568	0.029*	
H5B	0.205558	0.401856	0.281613	0.029*	
C6	0.2942 (2)	0.52788 (16)	0.23444 (8)	0.0175 (4)	
C7	0.6867 (2)	0.65812 (15)	0.54354 (8)	0.0154 (4)	
C8	0.8152 (2)	0.74659 (16)	0.54139 (8)	0.0182 (4)	
H8A	0.813831	0.778752	0.503793	0.022*	
H8B	0.783289	0.806039	0.567230	0.022*	
C9	0.9870 (2)	0.71586 (15)	0.55452 (8)	0.0154 (4)	
C10	0.8886 (2)	0.28840 (15)	0.44433 (8)	0.0175 (4)	
C11	1.0631 (2)	0.26360 (16)	0.45697 (9)	0.0233 (4)	
H11A	1.070206	0.234342	0.495073	0.028*	
H11B	1.099936	0.204546	0.431670	0.028*	
C12	1.1769 (2)	0.36074 (16)	0.45190 (9)	0.0203 (4)	
O1	0.35531 (19)	0.74834 (14)	0.32680 (6)	0.0303 (3)	
O2	0.02178 (18)	0.75665 (12)	0.21346 (6)	0.0244 (3)	
H2C	0.026 (4)	0.8130 (19)	0.1931 (11)	0.038 (8)*	
H2D	0.028 (5)	0.701 (2)	0.1947 (15)	0.080 (13)*	
O3	0.07710 (18)	0.87050 (11)	0.30898 (6)	0.0215 (3)	
O4	0.32172 (17)	0.86178 (11)	0.22910 (6)	0.0196 (3)	
O5	0.01295 (19)	1.04423 (12)	0.32610 (7)	0.0262 (3)	
O6	0.38542 (17)	1.03131 (12)	0.20276 (6)	0.0232 (3)	
O7	0.06645 (19)	0.63882 (11)	0.31152 (6)	0.0222 (3)	
O8	0.30314 (18)	0.63319 (11)	0.22957 (6)	0.0201 (3)	
O9	−0.01402 (18)	0.47315 (12)	0.33805 (6)	0.0251 (3)	
O10	0.34264 (18)	0.46437 (12)	0.19827 (7)	0.0243 (3)	
O11	0.9394 (2)	0.40964 (12)	0.56450 (7)	0.0301 (3)	
O12	0.91515 (18)	0.59625 (11)	0.43422 (6)	0.0205 (3)	
H12A	0.934 (4)	0.563 (2)	0.4037 (10)	0.046 (9)*	
H12B	0.974 (3)	0.6532 (18)	0.4332 (11)	0.031 (7)*	
O13	0.71826 (16)	0.55905 (11)	0.52868 (6)	0.0187 (3)	
O14	1.03772 (16)	0.61833 (11)	0.54409 (7)	0.0222 (3)	
O15	0.55026 (16)	0.68753 (11)	0.55807 (6)	0.0202 (3)	
O16	1.07776 (17)	0.78642 (11)	0.57380 (6)	0.0205 (3)	
O17	0.82840 (16)	0.38163 (11)	0.45893 (6)	0.0189 (3)	
O18	1.14289 (16)	0.45036 (11)	0.47688 (6)	0.0227 (3)	
O19	0.80826 (18)	0.21662 (12)	0.42110 (7)	0.0300 (4)	
O20	1.30257 (19)	0.34831 (13)	0.42501 (8)	0.0321 (4)	
N1	0.5604 (2)	0.49194 (15)	0.41104 (8)	0.0220 (3)	
H1A	0.576 (3)	0.490 (2)	0.3747 (8)	0.032 (7)*	
H1B	0.478 (3)	0.455 (2)	0.4191 (12)	0.041 (8)*	
H1C	0.561 (3)	0.5586 (16)	0.4223 (10)	0.022 (6)*	
H1D	0.639 (3)	0.463 (2)	0.4265 (12)	0.041 (8)*	
N2	0.7424 (2)	0.74351 (16)	0.32571 (9)	0.0261 (4)	
H2E	0.714 (4)	0.739 (2)	0.3587 (9)	0.044 (8)*	
H2F	0.717 (4)	0.8101 (18)	0.3140 (11)	0.034 (7)*	
H2G	0.844 (3)	0.730 (2)	0.3247 (12)	0.039 (8)*	
H2H	0.697 (4)	0.689 (2)	0.3082 (12)	0.045 (9)*	
N3	0.4637 (2)	0.73855 (16)	0.14276 (7)	0.0216 (4)	
H3A	0.549 (3)	0.753 (2)	0.1276 (11)	0.036 (7)*	
H3B	0.386 (3)	0.732 (2)	0.1208 (11)	0.044 (8)*	
H3C	0.443 (4)	0.792 (2)	0.1649 (11)	0.042 (8)*	
H3D	0.477 (4)	0.678 (2)	0.1577 (14)	0.057 (10)*	
N4	0.1713 (2)	0.47891 (16)	0.08469 (8)	0.0219 (4)	
H4A	0.097 (3)	0.438 (2)	0.0725 (10)	0.030 (7)*	
H4B	0.164 (4)	0.486 (3)	0.1196 (9)	0.055 (10)*	
H4C	0.259 (3)	0.450 (2)	0.0776 (12)	0.040 (8)*	
H4D	0.162 (4)	0.5452 (19)	0.0741 (12)	0.039 (8)*	
V1	0.22054 (4)	0.74995 (3)	0.28043 (2)	0.01603 (8)	
V2	0.93035 (4)	0.48827 (2)	0.51199 (2)	0.01528 (8)	

### Atomic displacement parameters (Å^2^)

**Table:**

	*U*^11^	*U*^22^	*U*^33^	*U*^12^	*U*^13^	*U*^23^
C1	0.0188 (9)	0.0191 (9)	0.0140 (9)	0.0013 (7)	0.0016 (7)	0.0008 (7)
C2	0.0204 (9)	0.0183 (9)	0.0268 (10)	−0.0046 (8)	0.0054 (8)	−0.0056 (8)
C3	0.0125 (7)	0.0168 (9)	0.0232 (10)	0.0006 (7)	0.0016 (7)	−0.0010 (7)
C4	0.0201 (9)	0.0174 (9)	0.0157 (9)	−0.0015 (7)	0.0002 (8)	−0.0007 (7)
C5	0.0244 (9)	0.0180 (9)	0.0296 (11)	0.0034 (8)	0.0073 (8)	0.0080 (8)
C6	0.0134 (8)	0.0167 (8)	0.0225 (10)	0.0015 (7)	0.0003 (7)	0.0012 (7)
C7	0.0152 (8)	0.0183 (8)	0.0128 (9)	0.0002 (7)	−0.0019 (7)	0.0019 (7)
C8	0.0147 (8)	0.0146 (8)	0.0255 (9)	0.0005 (7)	−0.0005 (7)	−0.0005 (8)
C9	0.0154 (8)	0.0185 (9)	0.0123 (9)	−0.0016 (7)	0.0006 (7)	0.0009 (7)
C10	0.0169 (9)	0.0157 (8)	0.0199 (10)	−0.0006 (7)	0.0007 (7)	−0.0003 (7)
C11	0.0165 (9)	0.0166 (9)	0.0369 (11)	0.0020 (8)	−0.0010 (8)	−0.0021 (8)
C12	0.0148 (9)	0.0186 (9)	0.0274 (11)	0.0016 (7)	−0.0009 (8)	−0.0022 (8)
O1	0.0312 (8)	0.0327 (8)	0.0268 (8)	0.0009 (7)	−0.0055 (6)	0.0020 (8)
O2	0.0320 (8)	0.0141 (7)	0.0273 (7)	0.0011 (6)	−0.0044 (6)	−0.0012 (6)
O3	0.0205 (7)	0.0180 (6)	0.0260 (8)	−0.0008 (6)	0.0079 (6)	−0.0024 (5)
O4	0.0231 (7)	0.0151 (6)	0.0207 (8)	−0.0029 (5)	0.0061 (6)	−0.0012 (5)
O5	0.0258 (7)	0.0197 (7)	0.0333 (9)	0.0047 (6)	0.0106 (6)	0.0028 (6)
O6	0.0244 (7)	0.0180 (7)	0.0272 (8)	−0.0030 (6)	0.0048 (6)	0.0033 (6)
O7	0.0242 (7)	0.0162 (6)	0.0263 (8)	0.0007 (6)	0.0094 (6)	0.0014 (5)
O8	0.0233 (7)	0.0152 (6)	0.0217 (8)	0.0013 (5)	0.0070 (6)	0.0013 (5)
O9	0.0283 (7)	0.0196 (7)	0.0272 (8)	−0.0057 (6)	0.0104 (6)	−0.0004 (6)
O10	0.0243 (7)	0.0208 (7)	0.0279 (8)	0.0039 (6)	0.0029 (6)	−0.0047 (6)
O11	0.0403 (9)	0.0234 (7)	0.0267 (8)	0.0059 (7)	−0.0047 (7)	0.0058 (6)
O12	0.0235 (7)	0.0164 (7)	0.0217 (8)	−0.0007 (6)	0.0036 (6)	−0.0007 (5)
O13	0.0159 (6)	0.0162 (6)	0.0240 (7)	−0.0005 (5)	0.0014 (5)	−0.0015 (5)
O14	0.0163 (7)	0.0183 (6)	0.0320 (8)	0.0022 (5)	−0.0069 (6)	−0.0066 (6)
O15	0.0155 (6)	0.0202 (6)	0.0248 (7)	0.0019 (6)	0.0032 (6)	0.0000 (5)
O16	0.0199 (6)	0.0188 (6)	0.0227 (7)	−0.0042 (6)	−0.0030 (6)	−0.0031 (5)
O17	0.0150 (6)	0.0155 (6)	0.0261 (8)	0.0005 (5)	−0.0015 (6)	−0.0023 (5)
O18	0.0151 (6)	0.0182 (7)	0.0347 (9)	0.0003 (5)	0.0003 (6)	−0.0062 (6)
O19	0.0230 (7)	0.0201 (7)	0.0468 (10)	0.0003 (6)	−0.0077 (7)	−0.0126 (7)
O20	0.0192 (7)	0.0267 (8)	0.0504 (11)	−0.0009 (6)	0.0117 (7)	−0.0093 (7)
N1	0.0209 (8)	0.0189 (8)	0.0262 (10)	−0.0001 (8)	−0.0030 (7)	−0.0015 (7)
N2	0.0274 (9)	0.0205 (9)	0.0304 (10)	−0.0005 (8)	0.0079 (8)	0.0002 (8)
N3	0.0238 (9)	0.0205 (9)	0.0206 (9)	0.0007 (7)	0.0053 (7)	−0.0004 (7)
N4	0.0195 (8)	0.0196 (9)	0.0264 (10)	−0.0006 (7)	0.0040 (7)	−0.0006 (7)
V1	0.01743 (15)	0.01364 (15)	0.01701 (16)	−0.00016 (12)	0.00290 (11)	0.00055 (13)
V2	0.01464 (14)	0.01294 (14)	0.01825 (16)	0.00112 (12)	−0.00192 (12)	−0.00076 (12)

### Geometric parameters (Å, º)

**Table:**

C1—O5	1.236 (2)	O1—V1	1.5892 (15)
C1—O3	1.273 (2)	O2—V1	2.3211 (15)
C1—C2	1.527 (3)	O2—H2C	0.84 (2)
C2—C3	1.511 (3)	O2—H2D	0.82 (2)
C2—H2A	0.9900	O3—V1	2.0097 (14)
C2—H2B	0.9900	O4—V1	2.0223 (14)
C3—O6	1.241 (2)	O7—V1	2.0072 (14)
C3—O4	1.283 (2)	O8—V1	1.9970 (14)
C4—O9	1.239 (2)	O11—V2	1.5899 (15)
C4—O7	1.284 (2)	O12—V2	2.2954 (15)
C4—C5	1.514 (3)	O12—H12A	0.85 (2)
C5—C6	1.513 (3)	O12—H12B	0.84 (2)
C5—H5A	0.9900	O13—V2	2.0076 (14)
C5—H5B	0.9900	O14—V2	1.9708 (14)
C6—O10	1.233 (2)	O17—V2	2.0098 (14)
C6—O8	1.282 (2)	O18—V2	2.0198 (14)
C7—O15	1.244 (2)	N1—H1A	0.891 (19)
C7—O13	1.279 (2)	N1—H1B	0.84 (2)
C7—C8	1.517 (3)	N1—H1C	0.851 (19)
C8—C9	1.515 (2)	N1—H1D	0.83 (2)
C8—H8A	0.9900	N2—H2E	0.83 (2)
C8—H8B	0.9900	N2—H2F	0.88 (2)
C9—O16	1.233 (2)	N2—H2G	0.86 (2)
C9—O14	1.279 (2)	N2—H2H	0.87 (2)
C10—O19	1.233 (2)	N3—H3A	0.82 (2)
C10—O17	1.284 (2)	N3—H3B	0.84 (2)
C10—C11	1.519 (3)	N3—H3C	0.85 (2)
C11—C12	1.516 (3)	N3—H3D	0.83 (2)
C11—H11A	0.9900	N4—H4A	0.85 (2)
C11—H11B	0.9900	N4—H4B	0.85 (2)
C12—O20	1.244 (3)	N4—H4C	0.83 (2)
C12—O18	1.274 (2)	N4—H4D	0.85 (2)
			
O5—C1—O3	123.34 (18)	C7—O13—V2	129.64 (12)
O5—C1—C2	118.28 (18)	C9—O14—V2	131.62 (12)
O3—C1—C2	118.37 (17)	C10—O17—V2	125.06 (13)
C3—C2—C1	114.38 (16)	C12—O18—V2	126.06 (13)
C3—C2—H2A	108.7	H1A—N1—H1B	110 (3)
C1—C2—H2A	108.7	H1A—N1—H1C	110 (2)
C3—C2—H2B	108.7	H1B—N1—H1C	116 (3)
C1—C2—H2B	108.7	H1A—N1—H1D	109 (3)
H2A—C2—H2B	107.6	H1B—N1—H1D	108 (3)
O6—C3—O4	122.49 (19)	H1C—N1—H1D	105 (3)
O6—C3—C2	119.12 (17)	H2E—N2—H2F	108 (3)
O4—C3—C2	118.35 (17)	H2E—N2—H2G	107 (3)
O9—C4—O7	122.19 (19)	H2F—N2—H2G	114 (3)
O9—C4—C5	118.58 (17)	H2E—N2—H2H	107 (3)
O7—C4—C5	119.17 (17)	H2F—N2—H2H	116 (3)
C6—C5—C4	118.78 (16)	H2G—N2—H2H	106 (3)
C6—C5—H5A	107.6	H3A—N3—H3B	114 (2)
C4—C5—H5A	107.6	H3A—N3—H3C	107 (3)
C6—C5—H5B	107.6	H3B—N3—H3C	108 (3)
C4—C5—H5B	107.6	H3A—N3—H3D	105 (3)
H5A—C5—H5B	107.1	H3B—N3—H3D	107 (3)
O10—C6—O8	122.34 (18)	H3C—N3—H3D	115 (3)
O10—C6—C5	119.35 (17)	H4A—N4—H4B	111 (3)
O8—C6—C5	118.22 (17)	H4A—N4—H4C	109 (3)
O15—C7—O13	122.43 (17)	H4B—N4—H4C	108 (3)
O15—C7—C8	117.08 (16)	H4A—N4—H4D	112 (3)
O13—C7—C8	120.42 (16)	H4B—N4—H4D	101 (3)
C9—C8—C7	119.27 (16)	H4C—N4—H4D	115 (3)
C9—C8—H8A	107.5	O1—V1—O8	100.52 (8)
C7—C8—H8A	107.5	O1—V1—O7	100.39 (8)
C9—C8—H8B	107.5	O8—V1—O7	88.76 (6)
C7—C8—H8B	107.5	O1—V1—O3	100.81 (8)
H8A—C8—H8B	107.0	O8—V1—O3	158.63 (6)
O16—C9—O14	120.68 (17)	O7—V1—O3	88.61 (6)
O16—C9—C8	119.39 (17)	O1—V1—O4	98.44 (7)
O14—C9—C8	119.90 (16)	O8—V1—O4	87.15 (5)
O19—C10—O17	122.13 (18)	O7—V1—O4	161.16 (6)
O19—C10—C11	118.31 (17)	O3—V1—O4	88.53 (6)
O17—C10—C11	119.54 (17)	O1—V1—O2	178.57 (7)
C12—C11—C10	115.54 (16)	O8—V1—O2	80.83 (6)
C12—C11—H11A	108.4	O7—V1—O2	80.07 (6)
C10—C11—H11A	108.4	O3—V1—O2	77.83 (6)
C12—C11—H11B	108.4	O4—V1—O2	81.12 (6)
C10—C11—H11B	108.4	O11—V2—O14	98.10 (8)
H11A—C11—H11B	107.5	O11—V2—O13	97.84 (8)
O20—C12—O18	122.63 (18)	O14—V2—O13	88.90 (6)
O20—C12—C11	118.47 (17)	O11—V2—O17	98.45 (7)
O18—C12—C11	118.85 (18)	O14—V2—O17	163.20 (6)
V1—O2—H2C	114 (2)	O13—V2—O17	91.68 (6)
V1—O2—H2D	108 (3)	O11—V2—O18	99.14 (8)
H2C—O2—H2D	110 (3)	O14—V2—O18	87.02 (6)
C1—O3—V1	126.35 (13)	O13—V2—O18	162.94 (6)
C3—O4—V1	125.80 (13)	O17—V2—O18	87.54 (6)
C4—O7—V1	127.70 (14)	O11—V2—O12	177.91 (7)
C6—O8—V1	128.79 (13)	O14—V2—O12	83.92 (6)
V2—O12—H12A	116 (2)	O13—V2—O12	82.74 (6)
V2—O12—H12B	117.2 (19)	O17—V2—O12	79.51 (6)
H12A—O12—H12B	104 (3)	O18—V2—O12	80.36 (6)
			
O5—C1—C2—C3	−130.1 (2)	O5—C1—O3—V1	176.46 (15)
O3—C1—C2—C3	51.0 (3)	C2—C1—O3—V1	−4.7 (3)
C1—C2—C3—O6	130.24 (19)	O6—C3—O4—V1	−175.44 (14)
C1—C2—C3—O4	−51.8 (2)	C2—C3—O4—V1	6.7 (2)
O9—C4—C5—C6	144.90 (19)	O9—C4—O7—V1	173.81 (15)
O7—C4—C5—C6	−37.8 (3)	C5—C4—O7—V1	−3.4 (3)
C4—C5—C6—O10	−139.71 (19)	O10—C6—O8—V1	175.81 (14)
C4—C5—C6—O8	43.6 (3)	C5—C6—O8—V1	−7.6 (3)
O15—C7—C8—C9	147.45 (18)	O15—C7—O13—V2	−172.31 (14)
O13—C7—C8—C9	−35.4 (3)	C8—C7—O13—V2	10.7 (3)
C7—C8—C9—O16	−151.89 (18)	O16—C9—O14—V2	−178.19 (13)
C7—C8—C9—O14	30.1 (3)	C8—C9—O14—V2	−0.2 (3)
O19—C10—C11—C12	144.3 (2)	O19—C10—O17—V2	164.68 (16)
O17—C10—C11—C12	−37.0 (3)	C11—C10—O17—V2	−13.9 (3)
C10—C11—C12—O20	−131.2 (2)	O20—C12—O18—V2	170.31 (16)
C10—C11—C12—O18	51.3 (3)	C11—C12—O18—V2	−12.3 (3)

### Hydrogen-bond geometry (Å, º)

**Table:**

*D*—H···*A*	*D*—H	H···*A*	*D*···*A*	*D*—H···*A*
O2—H2*C*···O9^i^	0.84 (2)	2.08 (2)	2.902 (2)	165 (3)
O2—H2*D*···O5^ii^	0.82 (2)	1.99 (3)	2.758 (2)	157 (4)
O12—H12*A*···O9^iii^	0.85 (2)	1.98 (2)	2.826 (2)	174 (3)
O12—H12*B*···O15^iv^	0.84 (2)	2.04 (2)	2.855 (2)	161 (3)
N1—H1*A*···O6^v^	0.89 (2)	1.97 (2)	2.833 (2)	164 (2)
N1—H1*B*···O20^vi^	0.84 (2)	1.96 (2)	2.787 (2)	168 (3)
N1—H1*C*···O16^vii^	0.85 (2)	1.88 (2)	2.711 (2)	164 (2)
N1—H1*D*···O17	0.83 (2)	2.02 (2)	2.851 (2)	175 (3)
N2—H2*E*···O16^vii^	0.83 (2)	2.01 (2)	2.818 (2)	161 (3)
N2—H2*F*···O10^viii^	0.88 (2)	1.96 (2)	2.826 (3)	170 (3)
N2—H2*G*···O7^iii^	0.86 (2)	2.18 (2)	3.006 (2)	159 (3)
N2—H2*H*···O6^v^	0.87 (2)	2.04 (2)	2.865 (2)	156 (3)
N3—H3*A*···O20^ix^	0.82 (2)	2.12 (2)	2.874 (2)	153 (3)
N3—H3*B*···O19^viii^	0.84 (2)	1.92 (2)	2.759 (2)	173 (3)
N3—H3*C*···O4	0.85 (2)	2.04 (2)	2.828 (2)	153 (3)
N3—H3*D*···O8	0.83 (2)	2.33 (3)	2.800 (2)	116 (3)
N4—H4*A*···O15^x^	0.85 (2)	1.98 (2)	2.809 (2)	164 (3)
N4—H4*B*···O5^ii^	0.85 (2)	2.10 (3)	2.767 (2)	135 (3)
N4—H4*B*···O10	0.85 (2)	2.43 (3)	3.105 (2)	136 (3)
N4—H4*C*···O14^xi^	0.83 (2)	2.05 (2)	2.872 (2)	169 (3)
N4—H4*D*···O19^viii^	0.85 (2)	2.09 (2)	2.885 (2)	156 (3)
C2—H2*B*···O10^viii^	0.99	2.59	3.235 (3)	123
C5—H5*A*···O6^v^	0.99	2.58	3.275 (3)	127
C5—H5*B*···O2^ii^	0.99	2.59	3.432 (3)	143
C8—H8*A*···O15^iv^	0.99	2.51	3.207 (2)	127

Symmetry codes: (i) −*x*, *y*+1/2, −*z*+1/2; (ii) −*x*, *y*−1/2, −*z*+1/2; (iii) *x*+1, *y*, *z*; (iv) *x*+1/2, −*y*+3/2, −*z*+1; (v) −*x*+1, *y*−1/2, −*z*+1/2; (vi) *x*−1, *y*, *z*; (vii) *x*−1/2, −*y*+3/2, −*z*+1; (viii) −*x*+1, *y*+1/2, −*z*+1/2; (ix) −*x*+2, *y*+1/2, −*z*+1/2; (x) −*x*+1/2, −*y*+1, *z*−1/2; (xi) −*x*+3/2, −*y*+1, *z*−1/2.
